# A Comprehensive Decision Support System for Pediatrics Immunization

**Published:** 2018-02

**Authors:** Mohammad Hossein REIHANI, Morteza BAGHERPOUR, Asma ERJAEE

**Affiliations:** 1. Dept. of Industrial Engineering, Iran University of Science and Technology, Tehran, Iran; 2. Allergy Research Center, Dept. of Pediatrics, Shiraz University of Medical Sciences, Shiraz, Iran

## Dear Editor-in-Chief

Healthcare issues, especially those relevant to immunization programme, have always been economically and socially important (1). The pediatric immunization problem received attentions to itself as a fundamental issue for healthcare systems. In addition, immunization programs have significant impacts on public health and can be considered as one of the most cost effective disease preventing actions (1, 2). Pediatrics vaccination scheduling consists of complex series of struggles from health insiders to be introduced appropriately. The process is time consuming and can be defective in manual types of operating. Its main goal is to make the most of immunization coverage for children, even if they might lose one or more doses and fall behind the baseline plan.

At such events, new individualized plans are needed based on child's individual vaccination history. This is very harder to deal with, when some changes, like need for such catch-up plans, are being considered. On the other hand, sometimes, new vaccine series are introduced and new baseline plans are needed. Additionally, the process of adopting immunization plans with such changes is a difficult sell and so many clinical considerations are needed, even when we exclude possible human errors through this process. The scope may be more understandable when pertains to a national programme with thousands of children being included. Counted always as an important immunization group, newborn children need to get the highest possible immunization quality. However, once a child falls behind the baseline schedule, per some statics, they often do not catch-up until close to school age, when it is most likely to miss the maximum immunization coverage (3). More interestingly, there exist some other issues before or after the programme itself; the vaccine vials are perishable commodities which need to be produced and consumed in a certain amount and period, respectively. Another issue is that this nationwide programme is costly. In this regard, immunization performance needs to be monitored and evaluated by a comprehensive framework during or after the execution.

Based on best of authors' knowledge, there is a lack of modeling in immunization plans, especially in pediatric vaccination. However, researches like Engineer et al. suggest a modeling procedure to deal with pediatric immunization problem and cases of catch-up plans (3). This work is developed later into a universal decision support tool for vaccination of various groups from newborn children to adolescents (4). Abrahams and Ragsdale presented a decision support tool for travel vaccines administration (5). There are other works, which try to provide solution approaches for healthcare problems with introducing decision support systems (DSS) (6, 7).

Two factors of cost-effectiveness and maximum protection (quality) are amongst the most important factors in this area (8). Therefore, a well-planned comprehensive vaccination guideline can be effective for both health insiders and immunization target groups. Thus, mathematical and quantitative methods and procedures can serve for such problems, either in economic or clinical terms. It seems there is a drastically crucial need for a comprehensive DSS, which makes decisions on so many issues from number of vials to produce, to immunization administration and its performance monitoring and evaluation. This planning approach will give a good quantitative perspective of vials inventory policies as perishable goods, an decision support tool which can deal individually when there exist needs for catch-up plans and finally a performance monitoring framework which can give a confined practical picture of how exactly good the programme performs. This is the structure of a comprehensive DSS with three main parts that each affects others. For instance, reports of the middle part, the catch-up rescheduling, will give some insights about vials usage and is useful in forecasting vials production.

Considering the previews DSSs to address immunization programme, still a comprehensive planning guideline, which considers pediatrics immunization programme from one step earlier of vaccination, which is vials production planning, and one step after that, performance monitoring and evaluation, is missing in the literature. It is mostly because the importance of both costs of immunization and highest possible immunization quality (8). In other words, in order to reduce health cost and maximize the quality, the desired comprehensive pediatric immunization planning procedure can involve three main parts. First, a vial inventory-production plan which is developed based on other two steps, second, the individualized scheduling and rescheduling decision support tool for possible deviations from the baseline plan and third, a performance evaluation framework with ability to monitor and forecast the whole immunization programme.
